# Bibliography

**Published:** 1921-01

**Authors:** 


### BIBLIOGRAPHY

BLOCK ANESTHESIA. Under the title of "Block Anesthesia," Dr. Arthur E. Smith has written the most comprehensive treatise on local anesthesia that has as yet been attempted. As it is a book of more than eight hundred pages, with six hundred illustrations, and has been in preparation for almost a decade, one can scarcely make an adequate review of such a work in a few lines. It is a splendidly made book in every way and the publishers have certainly done a most creditable piece of work.

Dr. Smith has for years been known for his enthusiastic interest in the subject of local anesthesia, and particularly with reference to dentistry. He has applied himself with such devotion that he has accumulated a fund of information that has made him the recognized authority on this subject. It is not, therefore, surprising that he could have produced a book that commands our admiration and makes us feel that we now have received a contribution to our literature that at once standardizes it and will in years to come be quoted in all medical and dental practice.

The illustrations are all original and are practically the result of the work of the author and from his own researches. The illustrations are none the less surprising than the completeness of the text. In both departments there is nothing, so far as we can see, that one feels has been slighted or inadequately treated. The illustrations are clearly drawn to assist in making the text free from redundant descriptions of technical procedures which word portrayals tend to confuse in the student mind. This is not a manual, but a serious endeavor to make an adequate technical statement of what is known of practical dental anesthesia, and as it is practiced by the most skillful dental surgeons of the day. We are confident that the book will commend itself to all students of dental surgery, and to the general and the special practitioners of dentistry, and to medical surgeons who may be interested in this phase of our art.

The profession is profoundly grateful to Dr. Smith for what he has done so thoroughly and scientifically. We are also most appreciative of the thorough and satisfactory publication of the book; it is an admirable specimen of book-making and is a credit to the publishers. Published by the C. V. Mosby Co., St. Louis. Price, $15.

